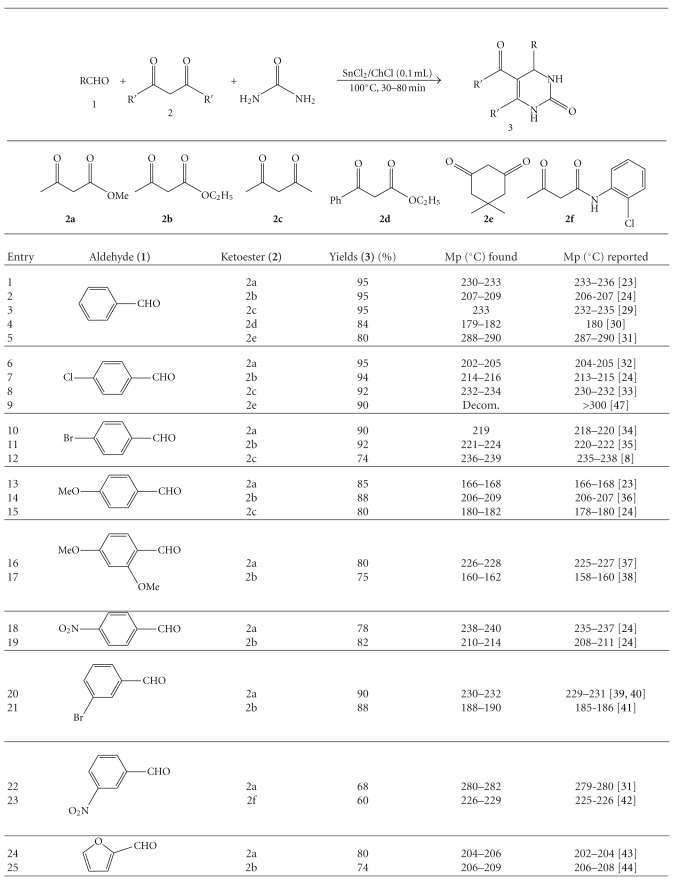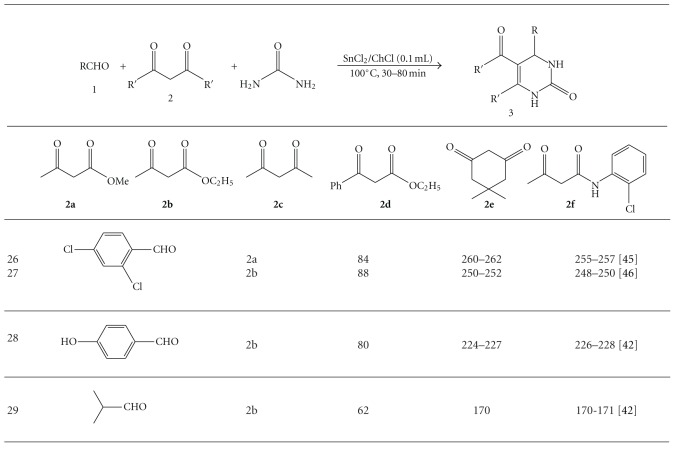# Eutectic Salt Catalyzed Environmentally Benign and Highly Efficient Biginelli Reaction

**DOI:** 10.1100/2012/908702

**Published:** 2012-04-29

**Authors:** Najmadin Azizi, Sahar Dezfuli, Mohmmad Mahmoodi Hahsemi

**Affiliations:** ^1^Chemistry & Chemical Engineering Research Center of Iran, P.O. Box 14335-186, Tehran 1496813151, Iran; ^2^Department of Chemistry, Science and Research Branch, Islamic Azad University, Tehran, Iran

## Abstract

A simple deep eutectic solvent based on tin (II) chloride was used as a dual catalyst and environmentally benign reaction medium for an efficient synthesis of 3,4-dihydropyrimidin-2(1H)-one derivatives, from aromatic and aliphatic aldehydes, 1,3-dicarbonyl compounds, and urea in good-to-excellent yields and short reaction time. This simple ammonium deep eutectic solvent, easily synthesized from choline chloride and tin chloride, is relatively inexpensive and recyclable, making it applicable for industrial applications.

## 1. Introduction

In recent years, utilization of room-temperature ionic liquids (RTILs) in organic synthesis and industry has received great attention due to their unusual properties compared with traditional molecular solvents, such as undetectable vapor pressure, wide liquid temperature range, special solubility for many organic or inorganic compounds, and favorable environments. A closely related class of solvents with physical properties and phase behaviors very similar to those of RTILs are room-temperature deep eutectic solvents (DESs), which were developed by Abbott and coworkers. These eutectic mixtures are attractive alternatives to RTILs, as DESs can be less expensive, more synthetically accessible, nontoxic, and biodegradable [1–4]. 

**Figure 1:**
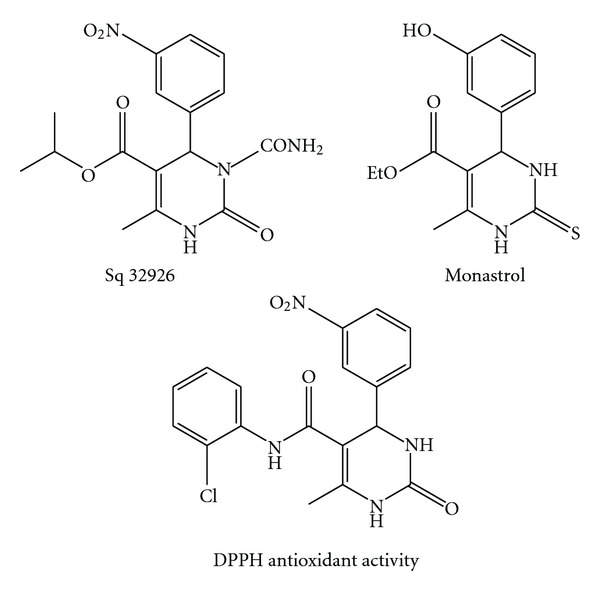
Some biologically active dihydropyrimidinones derivatives.

The Biginelli reaction is an important and one-pot, multicomponent domino reaction, which allows easy access to Polyfunctionalized dihydropyrimidinones (DHPMs) in an environmentally benign and atom-economic fashion of an aldehyde, urea, and a *β*-ketoester under strongly acidic conditions [5–8]. In recent years, dihydropyrimidinones and their derivatives occupy an important place in the realm of natural and synthetic organic chemistry because of their biological activities such as antiviral, antitumor, antibacterial, and anti-inflammatory properties. In addition, these compounds have emerged as potent calcium channel blockers, therapeutic and pharmacological properties (Figure 1) [9–12].

Due to their several applications in the biology and medical chemistry, considerable interest in this transformation has steadily increased over the past decade and several improved procedures have recently been reported [13–47]. However, some of the methods employed for DHPM synthesis have drawbacks, for example, the use of strongly acidic conditions, the use of protic acids, prolonged reaction times, low-to-moderate yields, and organic solvent.

## 2. Materials and Methods

### 2.1. Chemicals

Reactions were monitored by TLC and GC. FT-IR spectra were recorded using KBr disks on a Bruker Vector 22 FT-IR Spectrometer, ^1^H NMR spectra were recorded on 500 MHz NMR spectrometer, and ^13^C NMR spectra were recorded on 125 MHz NMR spectrometer, respectively, using CDCl_3_ or DMSO, as a solvent. Chemical shifts have been expressed in ppm downfield from TMS. Melting points were recorded on Buchi 535 melting point apparatus and are uncorrected. All starting materials and choline chloride and Tin chloride are commercially available and were purchased and used without further purification. Water and other solvent were distilled before used. 

#### 2.1.1. Preparation of Deep Eutectic Solvent

The general route for the synthesis of the ionic liquids was as follows: choline chloride (100 mmol) was mixed with tin chloride (200 mmol) and heated to ca. 100°C in air with stirring until a clear colourless liquid was obtained.^1^

#### 2.1.2. General Procedure

A mixture of benzaldehyde (3 mmol), urea (3 mmol), and methyl acetoacetate (3 mmol) in tin (II) chloride-choline chloride (2 : 1) (0.1 mL) ionic liquid was added into a test tube with a magnetic stirring bar under N_2_ atmosphere. The test tube was heated in an oil bath at 100°C for 30 minutes and then was cooled to room temperature slowly, and ethyl acetate and in some cases ethanol (10 mL) was added slowly and filtered off to extract the product from the deep eutectic solvent. For most of the reactions, purification was not necessary and the products were analyzed by ^1^H NMR spectroscopy and melting point; however, appropriate recrystallization in hot ethanol was used for further purification. All compounds were known and were characterized on the basis of their spectroscopic data (IR, NMR) and melting point by comparison with those reported in the literature.

## 3. Results and Discussion

During the course of our study aiming at improving the ecocompatibility of certain organic processes, we have been particularly interested in the development of organic transformations in a purely aqueous system to develop environmentally benign reactions [48–57]. Herein, we wish to report deep eutectic solvent as a novel catalyst and reaction medium for an efficient preparation of 3,4-dihydropyrimidinones under mild reaction conditions with short reaction time and simple work-up. 

**Scheme 1:**
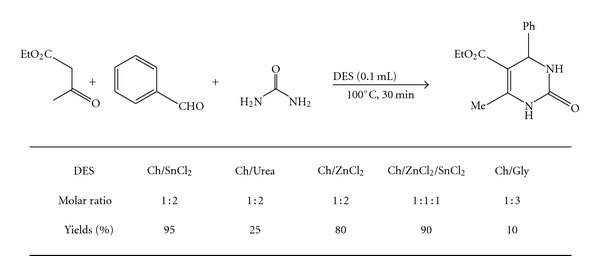
Optimization of reaction condition.

In an initial experiment, benzaldehyde (1 mmol) was treated with urea (1 mmol) and ethyl acetoacetate (1 mmol) in five choline-based deep eutectic solvents at different reaction condition. First findings indicated that, Tin chloride/choline-chloride-(ChCl/SnCl_2_-) based deep eutectic solvent (0.1 mL) at 100°C was an excellent reaction condition and dihydropyrimidinones (DHPMs) **3** were obtained in 95% yield in 30 min (Scheme 1). Other chlorine-based ionic liquids such as urea/choline chloride, polyethylene/choline chloride, zinc chloride/choline chloride drive the desired products in low yields. 

Under optimized reaction conditions, a broad range of structurally diverse 1,3-dicarbonyl compounds such as ethylacetoacetate **(2a)**, methylacetoacetate **(2b)**, pentane-2,4-dione **(2c)**, ethyl 3-oxo-3-phenylpropanoate, **(2d)** 5,5-dimethyl-1,3-Cyclohexanedione **(2e)**, 3-oxo-N-(2-chlorophenyl)butanamide **(2f)**, aromatic and aliphatic aldehydes, and urea are subjected to this green procedure to produce the corresponding dihydropyrimidinones not only in good yield but also with higher reaction rates (Table 1). A wide variation of alkyl groups and sensitive functionalities such as NO_2_,Cl,OH,OMe, and heterocyclic moieties in 1,3-dicarbonyl compound as well as in aldehyde are tolerated in this procedure to provide a library of dihydropyrimidinones with a variety of substituents.

In general, the reactions are very clean, and no side product was obtained in any run. In fact, the crude products obtained are of high purity and do not require any chromatographic separation in most cases. Recrystallization from hot ethanol provides analytically pure sample. Furthermore, deep eutectic solvent was recycled and reused for four times without any loss of activity.

## 4. Conclusion

This work describes the one-pot, three-component procedure of the synthesis of dihydropyrimidin-2(1H)-ones in deep eutectic solvent that provides an efficient and practical modification of Biginelli reaction. The separation and purification process are very simple and convenient, only needing recrystallization. Starting materials are inexpensive and commercially available. Moreover, this method offers several advantages including high yields, short reaction times, and a simple work-up procedure, and it also has the ability to tolerate a wide variety of substitutions in all three components, which is lacking in existing procedure.

## Figures and Tables

**Table 1:** (SnCl_2_)_2_ChCl catalyzed green synthesis of dihydropyrimidinons.